# Efficient long-term cryopreservation of pluripotent stem cells at −80 °C

**DOI:** 10.1038/srep34476

**Published:** 2016-10-03

**Authors:** Ye Yuan, Ying Yang, Yuchen Tian, Jinkyu Park, Aihua Dai, R. Michael Roberts, Yang Liu, Xu Han

**Affiliations:** 1 Division of Animal Sciences and Bond Life Sciences Center; University of Missouri, Columbia, MO 65211, USA; 2 Department of Mechanical & Aerospace Engineering; University of Missouri, Columbia, MO 65211, USA; 3 Comparative Medicine Center, University of Missouri, Columbia, MO 65211, USA

## Abstract

Current long term cryopreservation of cell stocks routinely requires the use of liquid nitrogen (LN_2_), because commonly used cryopreservation media containing cell membrane permeating cryoprotectants are thermally unstable when frozen at higher storage temperatures, e.g. −80 °C. This instability leads to ice recrystallization, causing progressive loss of cell viability over time under the storage conditions provided by most laboratory deep freezers. The dependency on LN_2_ for cell storage significantly increases operational expense and raises issues related to impaired working efficiency and safety. Here we demonstrate that addition of Ficoll 70 to cryoprotectant solutions significantly improves system thermal stability at the working temperature (~−80 °C) of laboratory deep freezers. Moreover, a medium comprised of Ficoll 70 and dimethyl sulfoxide (DMSO) in presence or absence of fetal bovine serum (FBS) can provide reliable cryopreservation of various kinds of human and porcine pluripotent stem cells at −80 °C for periods that extend up to at least one year, with the post-thaw viability, plating efficiency, and full retention of pluripotent phenotype comparable to that achieved with LN_2_ storage. These results illustrate the practicability of a promising long-term cryopreservation method that completely eliminates the need for LN_2_.

Pluripotent stem cells have an ability to self-renew, yet can also be induced to differentiate into a wide range of differentiated cell types. The first of these features means that such cells can provide an almost indefinite reserve of undifferentiated cells that can be cryopreserved for future use. The second is that pluripotent stem cells can be induced to differentiate into a wide range of mature cell types and provide a unique resource to study basic developmental processes and a largely untapped potential as a source of cells for tissue replacement and repair^1^,^2^. The ability to preserve stocks of quality-controlled lines of stem cells and to ship cryopreserved material safely and conveniently by air between different geographic locations at reasonable cost are important challenges to both small and large laboratory operations^3^,^4^.

Pluripotent stem cells come in two main types, although each may be convertible to the other^5^,^6^,^7^. The first, exemplified by those from the mouse, is the so-called “naïve” type, which is dependent upon leukemia inhibitory factor (LIF) and STAT3 signaling for growth. The second, typified by the human, monkey, and pig, is often named epiblast-type and requires FGF2 for self-renewal and maintenance of pluripotency. Whereas naïve type cells form domed colonies that can be readily dispersed into single cells for passaging and freezing, the latter form flat, adhesive colonies, and the cells lose viability when dissociated from each other unless special precautions are taken^8^,^9^. As a consequence, epiblast-type stem cells have historically been passaged and cryopreserved as clumps. However, there are limitations to freezing clumps, as cryoprotectant may penetrate the clump poorly so that, only a small fraction of the cells in the clump survive after cryopreservation. Plating efficiency is typically low and clonal propagation difficult^10^,^11^,^12^. More recently, addition of RHO-kinase (ROCK) inhibitors before freezing and after thawing has been demonstrated to improve cryopreservation efficiency and subsequent clonal growth of human ESC^13^,^14^,^15^,^16^,^17^.

Two approaches are widely used in cryopreservation: equilibrium (slow freezing) and non-equilibrium (vitrification) cooling procedures. The vitrification method^18^, as well as its “slow vitrification” variant^19^, not only introduces cell osmotic damage and toxicity due to the use of high concentrations (typically 40–50% v/v) of permeating cryoprotectant but requires LN_2_ or other cryogenic liquids to achieve and maintain vitrification of both intracellular and extracellular solutions at cryogenic temperatures, e.g. the saturation temperature of LN_2_ at one atmosphere pressure (−196 °C) or LN_2_ vapor (typically −120 °C). For slow freezing, cells are loaded with a low concentration (typically 10% v/v) of cryoprotectant and then slow-cooled to an intermediate temperature, e.g. −80 °C in a deep freezer^20^. During cooling, ice precipitation gradually increases solute concentrations, such that, after reaching the intermediate temperature, the residual solution containing the cells becomes highly concentrated and in a viscous liquid state^21^. The extracellular ice in such a partially frozen system is unstable, and the small ice crystals formed during cooling spontaneously begin to merge and form larger crystals to minimize their surface energy^22^,^23^. This so-called recrystallization phenomenon can cause mechanical damage to cells and also introduce lethal intracellular ice formation^21^,^24^. Even though the process is quite slow (typically occurring over weeks rather than hours), it is progressive, even at temperatures as low as −80 °C^25^,^26^,^27^,^28^,^29^. Accordingly, it is generally necessary to have a second step in which the samples are cooled from −80 °C to cryogenic temperatures.

However, long term storage of cell stocks through use of LN_2_ on an industrial or large laboratory scale typically requires cryogenic freezers, high pressure LN_2_ tanks, and LN_2_ driven programmable cooling machines, which are expensive to purchase and maintain. Additionally, storage on a smaller scale, especially in LN_2_ dewars depends on constant replenishment of LN_2_. The deployment LN_2_ also raises numerous safety and maintenance issues and places a considerable financial burden on users. Mechanical deep freezers, with their working temperature typically at −80 °C, provide an alternative to LN_2_ for many purposes, such as preserving stocks of microbes and viruses^30^, and storage of human tissues^31^, and work well as long as the freezers are equipped with power back-up and, temperature monitoring and alarm systems. However, even with such add-ons, deep freezers still potentially offer significantly improved operational and cost efficiencies compared to LN_2_ for many applications. Cryopreservation at −80 °C, if proven to be successful, eliminates many of the safety concerns associated with use of LN_2_, namely cold burn, LN_2_ tank explosion, and operator asphyxiation. It also avoids the need for routine topping up of storage tanks, requires less costly facilities and equipment for larger scale biobanking and utilizes simpler operational procedures. Accordingly, efforts are being made to use mechanical deep freezers for long-term cryopreservation of cell lines. In some cases, for example red blood cells (RBC) and peripheral hematopoietic stem cells, these efforts have proved to be relatively successful, possibly because of special biophysical, e.g. rheological, properties of the cell types that allow them to survive the usual negative effects of ice re-crystallization over the course of storage^32^,^33^,^34^,^35^. Other cell types, however, including embryonic stem cells, lose viability and display altered properties over the course of storage at −80 °C, so that LN_2_ is still required^25^,^26^,^27^,^28^,^29^. Biological antifreeze compounds, specifically antifreeze proteins and antifreeze glycoproteins, potentially have value in cryopreservation by promoting thermal hysteresis, inhibiting ice recrystallization, and possibly improving membrane structural integrity^36^,^37^,^38^, but outcomes with both beneficial and detrimental effects have been mixed, and application impeded by the limited commercial availability of such compounds. Various types of peptide and glycopeptide analogs of biological antifreeze compounds, synthetic polymers, and small molecules that are not peptide or polymer-based, have been demonstrated to be effective ice recrystallization inhibitors (IRI)^39^,^40^. Acceptable cell cryopreservation has been achieved at low IRI concentrations (depending on IRI types) and without using permeating cell cryoprotectant for some cell types, including human and sheep red blood cells^39^, human embryonic liver cells^40^, rat mesenchymal stem cells^41^,^42^, mononuclear cells from human umbilical cord blood cells^43^, among others. Cell cryopreservation methods that utilize IRI present a promising technology because they eliminate or reduce the need for permeating cryoprotectants, but, for the most part, they still depend on LN_2_ storage. Human RBC have been stored successfully at −80 °C in a medium containing low concentrations of synthetic, small molecule IRI and at a low glycerol concentration (15%)^44^,^45^. This protocol had the additional advantage of mitigating the influence of temperature fluctuations. To the best our knowledge, however, the use of such IRI has not been extended to the cryopreservation of cells other than RBC at −80 °C. To overcome this shortcoming, we have developed a method for the long term storage of pluripotent stem cells at the typical operating temperatures for standard laboratory deep freezers through use of a commonly available polysaccharide, Ficoll 70.

## Results

### Differential scanning calorimetry analysis of the thermal stability of cryoprotectant solutions with and without Ficoll 70

**Figure 1:**
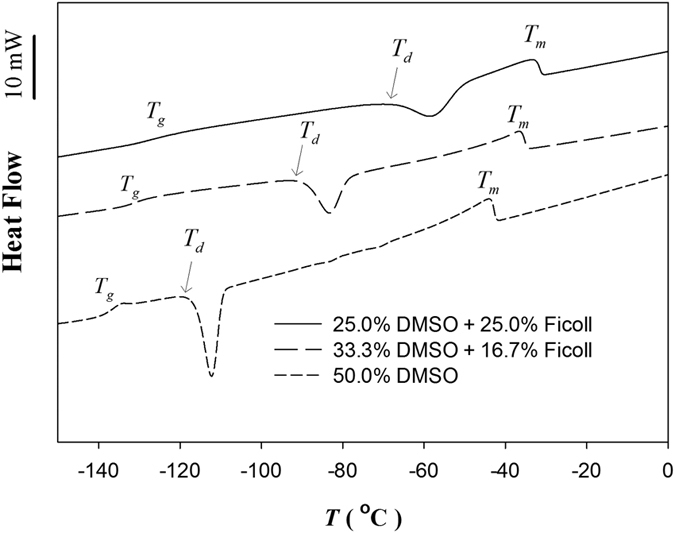
Examples of differential scanning calorimetric thermograms during the warming process of water-Ficoll 70-DMSO model solutions with different weight ratios of Ficoll 70 and DMSO and the same total solute ratio as 50% (w/w). The characteristic temperature transitions of these model solutions are marked: vitrification temperature (*T*_*g*_), devitrification temperature (*T*_*d*_) and melting point (*T*_*m*_). A scale bar (10 mW) is provided to evaluate the magnitude of heat flow during each phase transition stage.

To model the residual unfrozen portion of the cryoprotectant solutions at the end of a slow freezing process, highly concentrated ternary solutions were prepared. Exothermic curves were recorded during warming of these vitrified solutions, which had been pre-cooled to −160 °C, by using differential scanning calorimetry (see Fig. 1 for three examples). The model solutions tested in this manner were composed of a typical permeating cryoprotectant, DMSO and a non-permeating solute, namely Ficoll 70, Ficoll 400, polyvinylpyrrolidone (PVP) 40, PVP 360, and sucrose (Table 1). In all cases, total solute concentration was fixed as 50% w/w. For Ficoll, PVP, and sucrose, the solutions comprised an equal weight ratio of the DMSO and the non-permeating solutes, i.e. 25% w/v for each. The two additional model solutions tested were 50% DMSO and a mixture of 33.3% DMSO and 16.7% Ficoll 70 (Table 1; Fig. 1) to demonstrate the influence of the concentration of Ficoll 70. The thermal stabilities of each of these solutions were assessed by measuring their devitrification temperatures (*T*_*d*_) as demonstrated in Fig. 1. As shown in Table 1, Ficoll 70, with an equal weight of DMSO (25% Ficoll 70/25% DMSO), provided a higher *T*_*d*_ than any of the other solutes with the same amount of DMSO, and also performed better, i.e. provided a significantly higher *T*_*d*,_ than the 16.7% Ficoll 70/33% DMSO and 50% DMSO model solutions (Fig. 1). The 25% Ficoll 70/25% DMSO cryoprotectant solution increased the value of *T*_*d*_ to −67 °C, which is well above the storage temperature of a typical laboratory deep freezer. Since recrystallization temperature is always higher than *T*_*d*_^22^,^23^, we hypothesized that an approximately equal weight ratio of Ficoll 70 and DMSO has the potential to stabilize aqueous DMSO solutions when they are slowly cooled and then kept either in a −80 °C freezer or on a dry ice.

### Assessment of cell recovery of naïve-type O2K porcine iPSC after cryopreservation with freezing medium containing different concentrations of Ficoll 70

**Figure 2:**
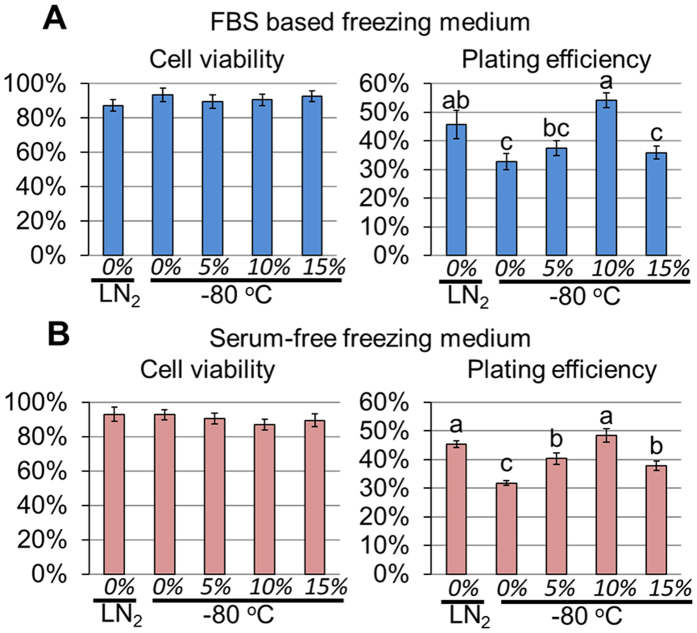
Assessment of cell recovery of naïve type O2K porcine iPSC cryopreserved with different concentrations of Ficoll 70 in FBS based (**A**) or serum-free DMEM/F12 based (**B**) medium to determine the optimal Ficoll composition. Data are for 2 weeks of storage. Within each figure, bar values are means ± SEM (n = 3), with different letters (a–c) indicating significantly different (P < 0.05) values. The prior-freezing samples of cell suspensions contained 10% (v/v) DMSO, various concentrations of Ficoll 70 (5%, 10% or 15%, w/v) for −80 °C storage. The samples containing 10% DMSO but without Ficoll were stored in either LN_2_ or in a −80 °C deep freezer and used for the two control groups.

Colonies of naïve-type porcine iPSC cells (O2K porcine iPSC)^46^ were dispersed by means of the commercial proteinase, Accutase©. The test media, based on either fetal bovine serum (FBS) or Dulbecco’s Modified Eagle Medium (DMEM, serum-free), were designed to provide a final concentration of 10% v/v DMSO and either 0%, 5%, 10% or 15% w/v Ficoll 70 after they had been added drop-wise to a suspension of cells in culture medium. After slow freezing to −80 °C, the vials were stored in a −80 °C freezer for two weeks. The control samples without Ficoll were cooled by the same slow freezing procedure and then stored either at −80 °C (as a negative control) or in a LN_2_ dewar (as a positive control). After thawing, only the cells cryopreserved at −80 °C with 10% Ficoll 70 provided a plating efficiency comparable to that of the LN_2_ storage control (Fig. 2). The lower (5%) and the higher (15%) concentrations of Ficoll afforded significantly worse survival, so that 10% was employed in all subsequent experiments. Importantly, the absence of FBS did not appear to exert a negative impact on cryopreservation.

### Assessment of cell recovery of naïve type O2K porcine iPSC after long term cryopreservation with Ficoll 70

**Figure 3:**
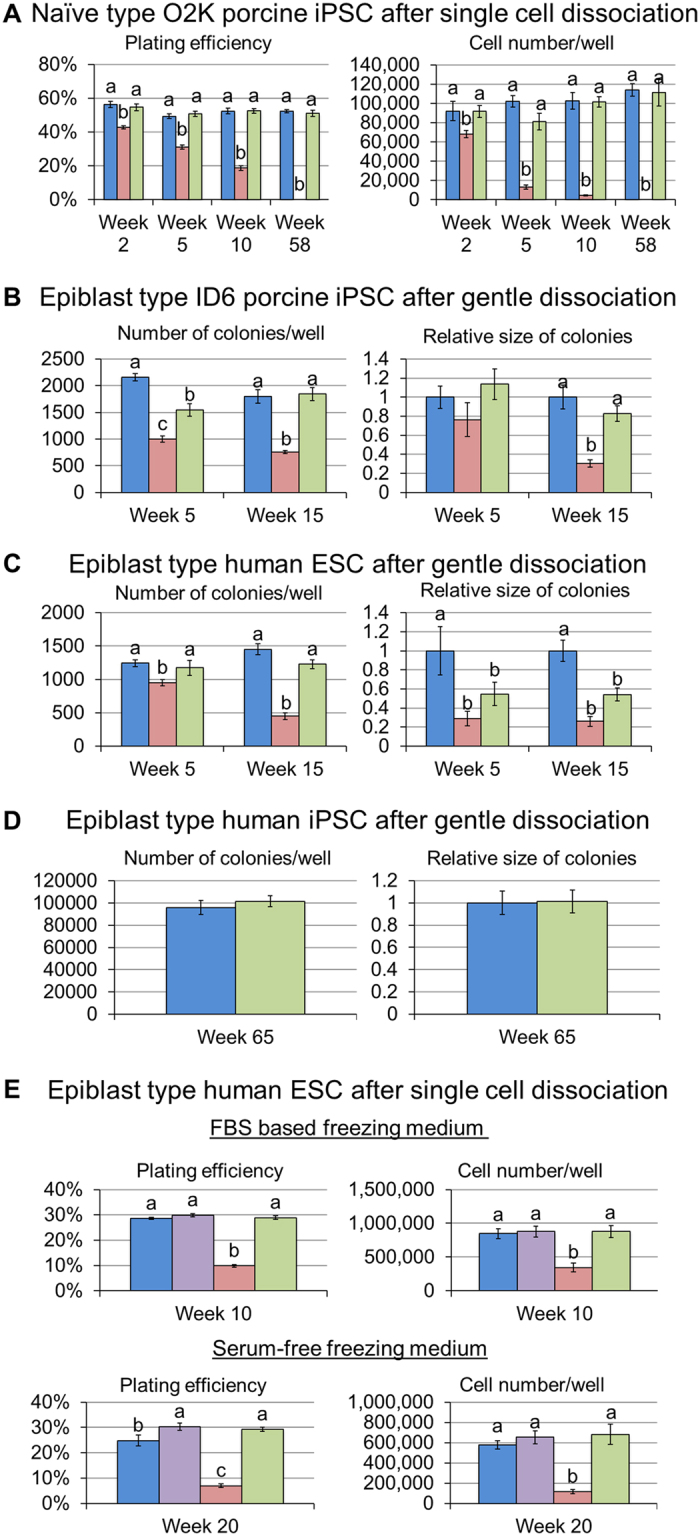
Post-thaw recovery of colonies from the naïve type O2K porcine iPSC (**A**), epiblast type ID6 porcine iPSC (**B**), epiblast type human H1 ESC (**C**,**E**) and epiblast type human iPSC (**D**) over extended storage periods. For naïve type cells (**A**), colonies were dispersed into single cells by Accutase. For epiblast type cells (**B**–**D**), cells were dispersed into small cell aggregates by Gentle Cell Dissociation Reagent. Human H1 ESC were also dispersed into single cells by TrypLE with the aid of ROCKi (**E**). Cells were cryopreserved under the following conditions: 10% v/v DMSO in the sample and stored in LN_2_ (blue); 10% v/v DMSO and10% w/v Ficoll 70 and stored in LN_2_ (purple); 10% v/v DMSO and stored in a −80 °C freezer (red); 10% v/v DMSO and 10% w/v Ficoll 70 and stored in a −80 °C freezer (green). Only FBS based freezing medium were used in (**A**–**D**). Both FBS based and serum-free freezing medium were tested in (**E**) Bar values are means ± SEM (n = 3), with different letters (a,b) indicating significantly different (P < 0.05) values within the results on the same checking point.

**Figure 4:**
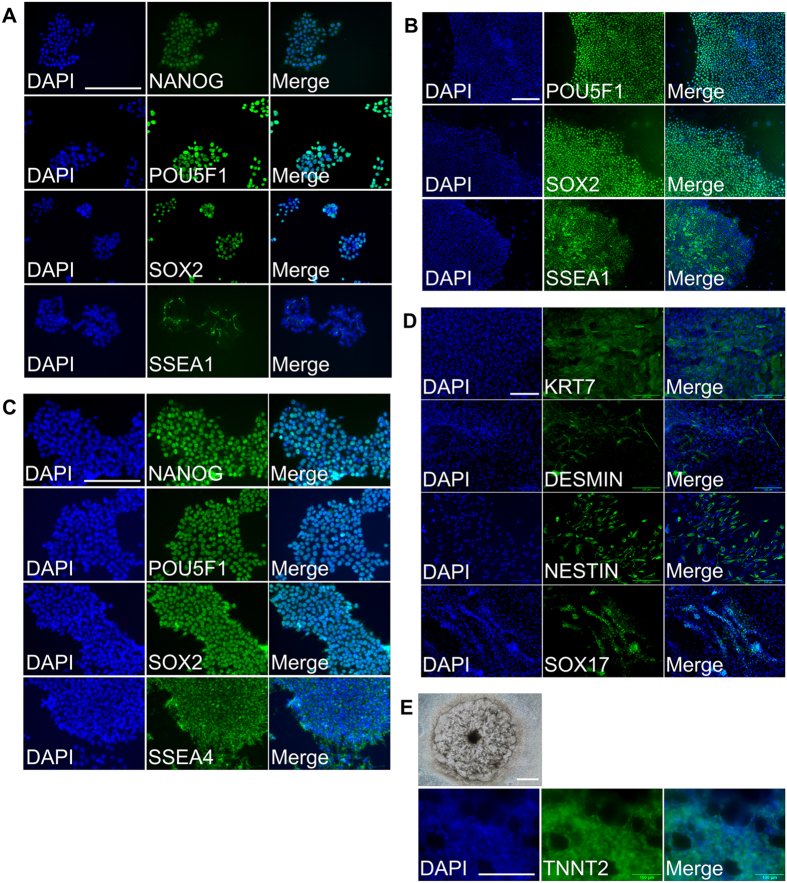
Pluripotent phenotypes of stem cells after recovery from cryopreservation in Ficoll 70 containing medium at −80 °C. (**A**–**C**), expression of pluripotent markers in O2K porcine iPSC (**A**), ID6 porcine iPSC (**B**), and H1 hESC (**C**) after recovery from cryopreservation in Ficoll 70- containing medium stored at −80 °C. (**D**) Lineage markers expressed in embryoid bodies differentiated from cryopreserved H1 hESC: KRT7 (trophectoderm), DESMIN (mesoderm), NESTIN (ectoderm), and SOX17 (endoderm). (**E**) Cardiomyocytes differentiated from cryopreserved H1 hESC: top panel, colony of beating cardiomyocytes; lower panels, expression of cardiac marker TNNT2. Scale bar = 200 μm.

Once an optimal concentration of Ficoll 70 in the freezing medium had been established, efficiency of cryopreservation was examined over more extended time periods. Vials from each treatment group were thawed after 2, 5, 10 weeks and 58 weeks. Even by week 2, the ability of the cells cryopreserved without Ficoll at −80 °C to attach and proliferate had fallen significantly relative to the other two treatments (Fig. 3A). These declines were progressive over storage time, such that at 10 weeks recovery was very low, and no colonies formed after 58 weeks of storage (Fig. 3A). By contrast, the porcine iPSC frozen at −80 °C with Ficoll-containing medium showed no apparent decrease in colony number (plating efficiency) and proliferative capacity (cell number) relative to LN_2_ storage over time (Fig. 3A). The expression of pluripotent markers NANOG, POU5F1, SOX2, and SSEA1 in these naïve type porcine cells was also not affected by cryopreservation (Fig. 4A).

### Assessment of cell recovery of epiblast type porcine iPSC, H1 human ESC (hESC) and human iPSC dissociated into small cell aggregates

**Figure 5:**
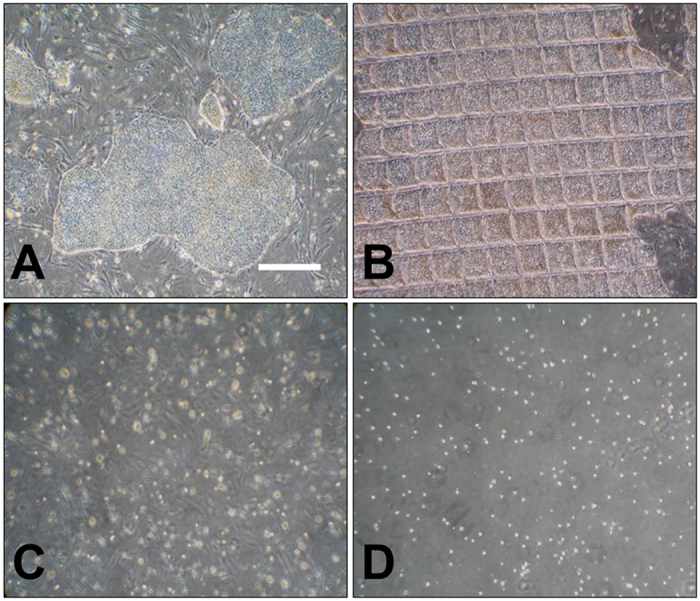
Dissociation of epiblast type stem cells by different methods. (**A**), morphology of epiblast type ID6 porcine iPSC during culture; (**B**), colonies manually cut by the cell passaging tool to yield uniform sized clumps; (**C**), colonies dispersed with Gentle Cell Dissociation Reagent for 6 min typically provided clumps of 6–8 cells; (**D**), colonies dispersed with TrypLE largely yielded single cells. Scale bar = 500 μm.

**Figure 6:**
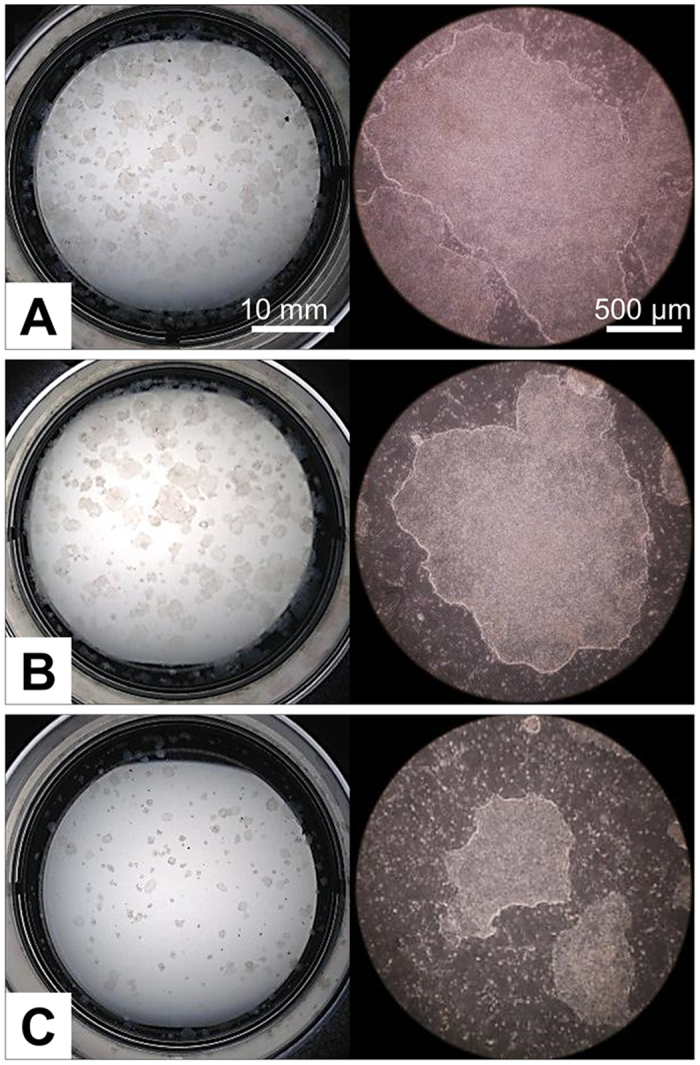
ID6 porcine iPSC colonies formed following 2 weeks of cryopreservation. Colonies were broken into uniform-size clumps (see Fig. 5B) by mechanical dissociation after dispase treatment and then cryopreserved. (**A**), LN_2_ without Ficoll 70; (**B**), −80 °C without Ficoll 70; (**C**), −80 °C with Ficoll 70. Images were taken at d 4 after thawing.

In the initial experiments with epiblast-type cells, ID6 porcine iPSC^47^ colonies were broken into large clumps (~100 cells) after dispase treatment and subsequent use of a cutting tool (Fig. 5A,B), which is the usual method of passaging the cells. In contrast to the naïve type cells, however, the cells stored at −80 °C in Ficoll-containing medium provided significantly fewer and smaller colonies (Fig. 6C) than ones from control medium without Ficoll 70 under both −80 °C and LN_2_ storage conditions (Fig. 6A,B). In other words, the presence of the Ficoll had negative consequences when large cell clumps were frozen. In the next experiment, ID6 porcine iPSC were dispersed prior to freezing by Gentle Cell Dissociation Reagent (STEMCELL Technologies) into small cell aggregates rather than being broken into large cell clumps (Fig. 5C). Samples were then thawed after 5 and 15 weeks storage. As observed with the naïve type porcine cells described earlier (Fig. 3A), the epiblast-type ID6 porcine iPSC cryopreserved in 10% Ficoll-containing medium at −80 °C provided a similar number of colonies per well (Fig. 3B, left panel) and similar colony sizes (Fig. 3B, right panel) after 15 weeks of storage as storage in LN_2,_ with no decline in cryopreservation efficiency (Fig. 3B). The 10% Ficoll 70 was also able to provide effective cryopreservation for human ESC (H1 cells) that had been dispersed by Gentle Cell Dissociation Reagent into small clumps (Fig. 3C, left panel). However, the sizes of the colonies recovered from the Ficoll-containing medium after −80 °C storage were smaller than those of the LN_2_ controls (Fig. 3C, right panel), even though colony number was the same. On the other hand, when a much larger number of human iPSC was employed, plating efficiency and colony size after 65 weeks of cryopreservation were no different for the cells stored in Ficoll-containing medium at −80 °C than for the LN_2_ controls (Fig. 3D).

The pluripotency markers (POU5F1, SOX2, and SSEA1 for ID6 porcine iPSC; NANOG, POU5F1, SOX2, and SSEA4 for H1 cells) continued to be expressed after thawing and further culture (Fig. 4). In addition, the thawed H1 ESC were able to form embryoid bodies that contained various types of differentiated cells characteristic of trophoblast and the three germ line lineages (Fig. 4D). It was also possible to drive differentiation of the thawed H1 cells to spontaneously contracting cardiomyocytes that expressed TNNT2, a marker of cardiac muscle (Fig. 4E and Supplementary Video 1).

### Cryopreservation of fully dispersed H1 hESC

We next used TrypLE dispersion of the H1 hESC colonies in presence of a RHO-kinase inhibitor (ROCKi, Y-27632) to achieve a suspension of single cells (Fig. 5D) that were stored under four test conditions (at −80 °C with and without 10% Ficoll 70 and in LN_2_ with and without Ficoll 70). The experiment was also replicated with FBS absent from the cryopreservation medium. Plating efficiency and total cell numbers were comparable between the cells kept at −80 °C with Ficoll 70 and LN_2_ controls (Fig. 3E). In addition, Ficoll 70 did not have a negative impact on LN_2_ storage. Moreover, as long as Ficoll 70 was present, cryopreservation remained efficient in absence of FBS (Fig. 3E). Again, cells cryopreserved at −80 °C without Ficoll 70 showed significantly reduced viability and proliferation relative to the other treatments (Fig. 3E).

**Figure 7:**
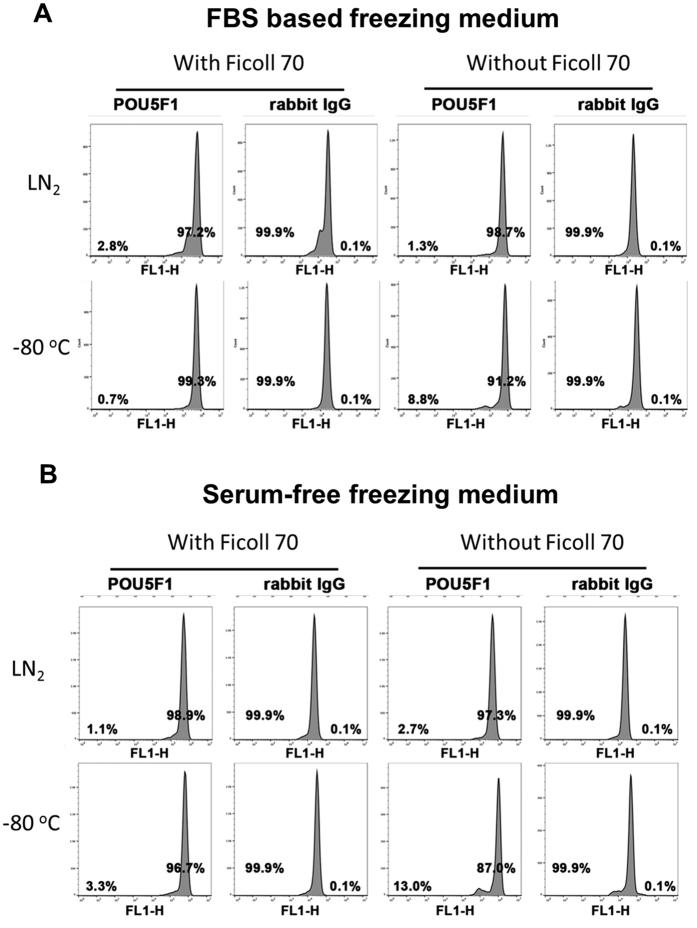
Flow cytometry histograms for POU5F1 expression in H1 hESC recovered from four different cryopreservation protocols (at −80 °C with and without Ficoll 70, and in a LN_2_ with and without Ficoll 70) after single cell dissociation by TrypLE with the aid of ROCKi. In the left panels (**A**), the cryopreservation medium was based on FBS; In the right panels (**B**), a second experiment utilized the same design but the FBS in the cryopreservation medium was replaced with the same volume of DMEM/F12. Values are means ± SEM (n = 3). At least 10,000 cells were analyzed for each sample. For the negative control, cells were exposed to rabbit IgG and a second antibody without prior exposure to primary antibody.

Thawed H1 cells were also analyzed by flow cytometry for the expression of the pluripotency marker POU5F1 (Fig. 7). Greater than 95% of the cells originating from LN_2_ storage and −80 °C storage with Ficoll 70 expressed POU5F1. Although a rather smaller percentage (~90%) of the cells originating from the −80 °C storage in absence of Ficoll 70 were positive for this marker, there was no indication that the majority had lost pluripotency, despite their poorer overall survival during cryopreservation.

## Discussion

The major goal of this research has been to develop a cryopreservation medium that is thermally stable at −80 °C, i.e. there is absence of recrystallization, in order to improve efficiency of long-term storage of stem cells in laboratory deep freezers, thereby eliminating the use of LN_2._ Such a medium would also be expected to ensure cell survivability during shipping and temporary storage on dry ice. To accomplish these goals, we developed a medium based on Ficoll 70, which, in aqueous solution, can restrict microscale diffusion and limit structural reconfiguration of macromolecules^48^,^49^. Ficoll 70 has been previously used for cryopreservation for several different cell types in LN_2_^50^,^51^,^52^,^53^ but was not selected for this purpose because of its ability to prevent recrystallization, as proposed here.

Because recrystallization is a spontaneous process that generates no detectable latent heat release^22^,^23^, the thermal stabilities of model cryoprotectant solutions could be assessed by measuring their devitrification temperatures (*T*_*d*_) (Table 1; Fig. 1). This approach is possible because, in cases when a solution is highly concentrated, *T*_*d*_ is always lower than, but close to the temperature at which recrystallization begins^22^,^23^. If the *T*_*d*_ measured during slow warming is higher than −80 °C, as observed for the solutions containing Ficoll 70 (*T*_*d*_, −67 °C) and Ficoll 400 (*T*_*d*_, −75.7 °C), it is considered to be thermally stable below that temperature. Therefore, no recrystallization will occur at −80 °C (Table 1). Of the two Ficoll polymers, Ficoll 70 was superior, since, at an equal weight ratio with DMSO, it demonstrated the higher *T*_*d*_ value.

As the field of regenerative medicine moves closer to the translational phase, there is an increased need for animal models, such as the pig, whose physiology and anatomy resemble those of the human better than rodents^54^,^55^,^56^,^57^, to establish protocols for stem cell-based regenerative medicine. Therefore, the cryopreservation efficiency of the Ficoll-containing medium was tested not only in hESC, but also for two kinds of porcine iPSC, one of the naïve type (O2K porcine iPSC)^46^, the other of the epiblast type (ID6 porcine iPSC)^47^. One big advantage of naïve-type cells is that they can be readily propagated from single cells after cryopreservation, so that cell viability after thawing can be readily measured. The experiments illustrated in Figs 2 and 3A demonstrate that the medium containing 10% Ficoll 70 can preserve naïve type porcine iPSC at −80 °C as efficiently as cryopreservation in LN_2_ for periods of more than one year, while cells stored frozen without Ficoll 70 at −80 °C rapidly deteriorate in quality as evidenced by a progressive decrease in plating efficiency and cell number, presumably due to the progressive generation of larger ice crystals as storage time increased^24^,^58^.

The Ficoll 70 medium also proved useful for the cryopreservation of epiblast-type cells of porcine and human origin as long as the colonies were dispersed into small clumps or single cells before freezing. However, even modest-sized clumps obtained after exposing the colonies to dispase and subsequent use of a cutting tool (Fig. 5B), which is the standard method of passaging such cells in many laboratories^59^, did not respond well to −80 °C storage in the Ficoll 70 cryoprotectant (Fig. 6). We suspect that the Ficoll, as a strong diffusion modifier, limited the diffusion of DMSO into the clumps (Fig. 5B). A potential solution is to alter the order of mixing procedure, for example, adding DMSO to the cell suspensions before the Ficoll. When H1 hESC were dispersed by Gentle Cell Dissociation Reagent, the sizes of the colonies recovered from the Ficoll-containing medium after −80 °C storage were smaller than those of the LN_2_ controls (Fig. 3C, right panel). We infer that larger size colonies are formed from clumps containing more viable cells, while the smaller colonies arose through death of some but not all of the cells in the clumps, a phenomenon that did not occur as readily during LN_2_ cryopreservation. As an alternative to the gentle dissociation procedure, colonies of H1 hESC can be fully dissociated into single cells by TrypLE. Then, provided that they are thawed and initially cultured in presence of a ROCK inhibitor, the cells are protected from apoptosis, and high freezing efficiency at −80 °C in the Ficoll 70 medium is achieved.

In conclusion, we have developed a simple method for long term storage of human and porcine pluripotent stem cells at −80 °C, based on the use of Ficoll 70, a synthetic polymer of sucrose. Since the addition of 10% Ficoll 70 had no negative impact on standard LN_2_ storage, its presence in the cryopreservation medium provides an extra bonus by minimizing the re-crystallization phenomenon, should the samples subsequently be shipped on dry ice. We infer that the success of the method is attributable to the ability of the Ficoll to improve the thermal stability of the permeating cryoprotectant solutions in the working temperature range of deep freezers. Additionally, the method can avoid the inclusion of FBS in the cryoprotectant solution, which is undesirable if the cells are planned for clinical application. Finally, even though we have focused on the cryopreservation of pluripotent stem cells, there is no reason to suppose that a Ficoll 70-containing medium will not provide efficient −80 °C freezing and long term storage for many other kinds of cells.

## Methods

### Measurement of the devitrification temperatures of highly concentrated model solutions by using differential scanning calorimetry

A standard, differential scanning calorimetry procedure was used to detect vitrification and devitrification (Pyris Diamond Differential Scanning Calorimeter, Perkin-Elmer Corp)^22^. A volume of 8 μl of each model solution (solutes purchased from Sigma-Aldrich) was sealed in a standard 10 μl aluminum crucible (Perkin-Elmer Corp) designed for liquid samples and then loaded in the sample holder of the calorimeter. All samples were cooled to −160 °C from 1 °C at 100 °C/min to achieve complete vitrification, which was confirmed by continuous heat capacity change near −130 °C for all the samples during the cooling and subsequent warming procedures. None of the samples experienced any crystallization during the cooling process. After being held at −160 °C for 1 min, the samples were heated to 20 °C at a warming rate of 10 °C/min. Devitrification was detected in all the samples. Onset temperatures for the corresponding exothermic curves were determined as the values of devitrification temperatures by using the Pyris™ thermal analytic software provided by Perkin-Elmer Corp.

### Cell sources and human subjects

The naïve-type O2K porcine iPSC^46^ and epiblast type ID6 porcine iPSC^47^ were generated in our laboratory. The human iPSC line was derived from human umbilical cord fibroblasts reprogrammed with five factors (*POU4F1*, *SOX2*, *KLF4*, *LIN28*, and *MYCL*) and *TP53* shRNA by using episomal plasmid transfection^60^ as described by Lee *et al*.^61^. Human umbilical cord tissues were collected, with informed consent, in University Hospital (University of Missouri) with approval from the University of Missouri Health Sciences Institutional Review Board (no. 1201132). Epiblast type H1 hESC (WA01) were obtained from the WiCell Research Institute, Madison WI in 2002. All experiments were performed in accordance with relevant guidelines and regulations.

### Routine maintenance of pluripotent stem cells

For routine maintenance, O2K porcine iPSC were cultured either on a laminin (Gibco) coated substratum or irradiated mouse embryonic fibroblasts feeder on six-well culture plates (Nunc) in N2B27 (Gibco) medium, supplemented with three inhibitors (CHIR99021(Stemgent), PD032591 (Stemgent), and PD173074 (Sigma)), 2 μg/ml doxycycline (Stemgent), and 1000 unit/ml human LIF (Millipore). O2K porcine iPSC were passaged every three days after dispersing with Accutase (Millipore) for 7 min at 37 °C. Epiblast type ID6 porcine iPSC were cultured on irradiated mouse embryonic fibroblasts (iMEF) feeder layers in six-well culture plates in standard hESC medium (hESCM) supplemented with 20% v/v knockout serum replacement (KOSR, Gibco) and 4 ng/ml human FGF2^62^,^63^. Human iPSC and H1 hESC were cultured on six-well culture plates (Nunc) coated with Matrigel (BD Bioscience) in defined mTeSR1 medium (STEMCELL Technologies). For epiblast-type ID6 porcine iPSC, human iPSC and H1 hESC, cells were passaged as clumps at a 1:6 ratio every 5–6 d by using the Stempro EZpassage (Invitorgen) cutting tool after dispase (1 mg/ml; STEMCELL Technologies) treatment for 7 min at 37 °C. All cells were cultured at 37 °C in a 5% O_2_, 5% CO_2_, and 90% N_2_ atmosphere with daily exchange of fresh medium.

### Freezing and thawing of cells

Dissociated cells were collected by centrifugation (200 × g for 5 min) and resuspended in chilled culture medium. For H1 hESC that had been dispersed by using TrypLE and frozen as single cells, 10 μM ROCK inhibitor (Y-27632) was included in the culture medium during freezing. Doubled target concentrations of Ficoll 70 were dissolved in either FBS or DMEM and then mixed with DMSO in a volume ratio of 5:1 to prepare the freezing media. Cell suspensions were mixed with freezing medium in a final volume ratio of 2:3 by slowly adding the freezing medium to the cell suspension. The cells and freezing medium were dispensed into cryovials (Nunc), and immediately placed into a freezing box (Mr. Frosty, Nalgene). The latter was placed overnight into an −80 °C freezer to provide an approximately 1 °C/min cooling rate. On the following day, the vials were placed in either LN_2_, or at −80 °C for storage. For thawing, cryovials were rapidly warmed in a 37 °C water bath for approximately 1 min until the ice disappeared. The cell suspension was then transferred to a 15 ml centrifuge tube and slowly mixed with 5 ml of warmed culture medium. After centrifugation (200 × g for 5 min), the supernatant solution was removed, and cell pellets resuspended in 1 ml fresh culture medium. Cells were plated in a 6-well plate and cultured in the conditions described above.

### The post-thaw recovery assessment for fully dispersed naive type O2K porcine iPSC

Thawed cells from three samples in each treatment group were transferred to a laminin-coated, 6-well plate and cultured overnight. After the first medium change, images of adherent colonies were acquired over five different areas within each well. Plating efficiency was estimated as colonies/number of initially plated cells ×100%. Colonies were then fully dispersed by Accutase, and total cell numbers were assessed by using a TC10 automated cell counter (Bio-Rad).

### The post-thaw recovery assessment for epiblast type ID6 porcine iPSC, human iPSC and H1 hESC dissociated into small cell aggregates

Thawed cells from three samples in each treatment group were transferred to 6-well plates coated with Matrigel (human iPSC hESC) or iMEF (ID6 porcine iPSC), with cells from one vial divided equally between two wells). On day 4 after thawing and plating, five images of different areas of each culture well were captured at 40× magnification to determine colony areas relative to the control group that had been stored in LN_2_. Cells were then fixed in 4% v/v paraformaldehyde in phosphate-buffered saline (PBS, Hyclone) for 2 min and stained for alkaline phosphatase activity to increase contrast. Nine images were taken at 8× magnifications to cover the entire area of the well and used to measure the total number of colonies present. All images were analyzed by the Image J software^64^.

### The post-thaw recovery assessment for fully dispersed epiblast type H1 hESC

H1 hESC colonies, after being dispersed into single cells prior to cryopreservation, were seeded into 6-well plates as described earlier, except the hESCM used had been previously conditioned by iMEF feeder and contained 10 μM Y-27632 in both the culture medium and the Matrigel. After cells had been resuspended in 1 ml fresh culture medium, 10 μl of the cell suspension was mixed with an equal volume of 0.4% Trypan blue dye (Bio-Rad) to assess cell viability. The number of blue cells, which were presumed to have experienced plasma membrane damage, and total cell numbers were assessed by using the TC10 automated cell counter (Bio-Rad). Plating efficiency was assessed on the second day after the first medium change. Images of adherent colonies were acquired over five different areas within each well. Plating efficiency was estimated as colonies/number of initially plated cells ×100%. Cultures were allowed to continue for another day when the colonies were fully dispersed by TrypLE, and cells were counted before being fixed for flow cytometry analysis.

### Immunocytochemistry

Thawed cells were allowed to establish colonies, passaged and grown on coverslips. Specimens were fixed in 4% v/v paraformaldehyde in PBS for 15 min at room temperature, washed, and exposed to either 5% v/v goat serum or 5% v/v donkey serum, 1% w/v bovine serum albumin, and 0.1% v/v Triton X-100 (Fisher) in PBS for 30 min. The fixed specimens were then incubated with primary antibodies at 4 °C overnight. After washing, they were exposed to secondary antibodies. Colonies exposed only to secondary antibody served as controls. VECTASHIELD mounting medium with DAPI (Vector Laboratories) was used to mount the coverslips. Primary antibodies were: POU5F1 (1:100; Santa Cruz Biotechnology, catalog no. sc-9081), SOX2 (1:1000; R&D Systems, catalog no. MAB2018), NANOG (1:200; Abcam, catalog no. ab109250), SSEA1 (1:50; Developmental Studies Hybridoma Bank [DSHB], Hybridoma Product MC-480 deposited by Solter, D./Knowles, B.B), SSEA4 (1:50; DSHB, Hybridoma Product MC-813-70 deposited by Solter, D./Knowles, B.B), KRT7(1:100; DAKO, catalog no. M701801-2), DESMIN (1:100; Santa Cruz Biotechnology, catalog no. sc-14026), NESTIN (1:100; Abcam, catalog no. ab6320), SOX17 (1:100; R&D Systems, catalog no. AF1924), TNNT2(1:100; Santa Cruz Biotechnology, catalog no. sc-8121). Images were taken under an Olympus IX70 inverted microscope (www.biotech.missouri.edu/mcc/Olympus.html).

### Spontaneous differentiation of hESC via embryoid body (EB) formation

Colonies of hESC were dispersed by dispase/mechanical dissociation and transferred into EB differentiation medium, consisting of DMEM/F12, 15% FBS, 1% non-essential amino acids, 1 mM L-glutamine, and 0.1 mM 2-mercaptoethanol, in low attachment plates (Corning). After 5 days of growth in suspension, the EB were seeded onto gelatin-coated plates and cultured in the same medium for another 9 days before fixation for immunocytochemistry.

### Directed differentiation of hESC into cardiomyocytes

The hESC were differentiated into cardiomyocytes by using Cardiomyocyte Differentiation Kit (Gibco) and following the manufacturer’s instructions. In brief, the hESC colonies, cultured on the Matrigel (BD Bioscience) coated plates in mTeSR1 medium (STEMCELL Technologies), were exposed to Cardiomyocyte Differentiation Medium A (Gibco) for two days, followed by Cardiomyocyte Differentiation Medium B (Gibco) for another two days. The cells were then cultured in the Cardiomyocyte Maintenance Medium for a further 8 days, when spontaneously contracting cardiomyocytes appeared. The expression of cardiomyocyte marker TNNT2 was confirmed by immunocytochemistry.

### Flow cytometry

The hESC were dispersed into single cells by TrypLE (Invitrogen) treatment for 7 min at 37 °C, fixed in a Foxp3 Fixation/Permeabilization solution (eBioscience) for 1 h on ice, and incubated in 5% (v/v) donkey serum for 15 min to reduce any nonspecific binding of antibodies. Cell were then exposed to an antibody directed against POU5F1 (1:200, Santa Cruz Biotechnology, catalog no. sc-9081) or to IgG (0.4 μg/mL; Santa Cruz Biotechnology, catalog no. sc-66931) in the blocking buffer for 1 h. All the steps were performed in the dark on ice, and cells were washed by Permeabilization Solution (eBioscience) three times between each step. For each cell population, at least 10,000 cells were analyzed in the Accuri C6 Flow Cytometer (BD Biosciences). Data were analyzed by the FlowJo (version X) software.

### Statistical analysis

Data were analyzed by using one-way ANOVA by means of the Software NCSS 2007 Version 07.1.4, with treatment included in the model as a fixed factor. For each experiment, comparisons of treatments were made at each of the time points. Percentage data were normalized by arcsin transformation. Differences were determined by Fisher LSD multiple comparison test when treatment was significant. Significance was categorized as *P* < 0.05. Data were reported as means ± SEM.

## Additional Information

**How to cite this article**: Yuan, Y. *et al*. Efficient long-term cryopreservation of pluripotent stem cells at −80 °C. *Sci. Rep*. **6**, 34476; doi: 10.1038/srep34476 (2016).

## Supplementary Material

Supplementary Information

Supplementary Video S1

## Figures and Tables

**Table 1:** The devitrification temperatures (*T*_*d*_) of the concentrated solutions modeling the unfrozen residual portion of aqueous solutions containing one polymer (or sucrose) and DMSO at the end of a slow freezing process.

Solute concentrations (w/w)	*T*_*d*_
25% Ficoll 70, 25% DMSO	−67.0 °C
25% Ficoll 400, 25% DMSO	−75.7 °C
25% PVP 40, 25% DMSO	−91.5 °C
25% PVP 360, 25% DMSO	−101.8 °C
25% sucrose, 25% DMSO	−110.1 °C
50% DMSO	−118.2 °C
16.7% Ficoll 70, 33.3% DMSO	−90.6 °C

The total solute weight percentage for each solution was fixed at 50% w/w.
