# Supplementary material for: Efficient long-term cryopreservation of pluripotent stem cells at −80 °C
Source: Sci Rep. 2016 Oct 3;6:34476. doi: 10.1038/srep34476 (PMC5046093; doi:10.1038/srep34476)
Supplement: Supplementary Information [file srep34476-s1.doc]

**Supplementary information for**

**Efficient long-term cryopreservation of pluripotent stem cells at -80 ^o^C**

**Ye Yuan, Ying Yang, Yuchen Tian, Jinkyu Park, Aihua Dai, R. Michael Roberts, Yang Liu, Xu Han**

Video legends

**Supplementary Video 1** Spontaneous contraction of cardiomyocytes differentiated from H1 hESC that were stored in –80 ^o^C in Ficoll-containing medium.
